# Serial C-reactive Protein Monitoring in Prosthetic Joint Infection: A Powerful Predictor or Potentially Pointless?

**DOI:** 10.7759/cureus.6967

**Published:** 2020-02-12

**Authors:** Rafia Ghani, Jonathan Hutt, Philip Mitchell, Luke Granger, Nemandra A Sandiford

**Affiliations:** 1 Orthopaedics, Russells Hall Hospital, Dudley, GBR; 2 Orthopaedics, St. George's University Hospital, London, GBR

**Keywords:** arthroplasty, orthopaedics, infection, prosthetic, prosthetic joint, revision, blood testing, inflammatory

## Abstract

Background

Serum C-reactive protein (CRP) is an important test in the initial diagnosis of prosthetic joint infection (PJI). There is no widely accepted algorithm for the resolution of PJI. Surgeons have traditionally used CRP to determine if the infection has resolved. However, this practice is not currently supported by significant data.

Methods

A retrospective analysis of our departmental arthroplasty database was conducted to determine mean values of CRP pre and postoperatively for PJI treated with the debridement, antibiotics and implant retention (DAIR) procedure, single-stage revision and two-stage revision. Receiver operating characteristic (ROC) curves were calculated to determine the sensitivity and specificity of CRP testing in diagnosing persistent infection.

Results

Of the 121 patients who had undergone treatment (75 hip replacements and 48 knee replacements), there were 26 cases of persistent infection. There was no statistical significance in the mean CRP values between successful and unsuccessful treatment groups. The areas under ROCs (AUCs) for CRP values predicting outcomes ranged from 0.46 to 0.73.

Conclusion

Our study does not support the use of serial CRP monitoring as an indicator of the successful eradication of PJI.

## Introduction

Prosthetic joint infection (PJI) is a serious complication of total joint arthroplasty of the hip and knee. It is estimated that 1-2.5% of patients undergoing primary total joint replacement require treatment for PJI [1]. There are well-established evidence-based algorithms in place for the initial diagnosis of PJI with considerable evidence to support the use of serum inflammatory markers in diagnosis [2]. Following the diagnosis of PJI, the main treatment modalities are debridement, antibiotics and implant retention (DAIR), single-stage revision or two-stage revision. Two-stage revision remains the gold standard of treatment for PJI [3]. In all treatment modalities, there is no widely accepted algorithm to determine infection resolution and the success of treatment. Many surgeons use serial serum C-reactive protein (CRP, an inflammatory marker) monitoring to determine response to treatment. However, there is no reliable evidence yet to suggest that low or decreasing CRP values indicate the elimination of infection.

Current evidence suggests that serial CRP monitoring cannot reliably determine infection control in two-stage revision; however, the role of CRP in assessing the success in DAIR and single-stage revision procedures remains unclear. Ghanem et al. studied 109 patients who had undergone two-stage revision for infected knee replacements from 1999 to 2006. They analysed the effectiveness of CRP as a test in determining the eradication of infection by using the area under a receiver operator characteristic (ROC) curve (AUC). They found the AUC for CRP to be 0.55, which was not statistically significant. The study concluded that CRP often does not normalise even when the infection is eradicated [4]. This conclusion was supported by Shukla et al. who retrospectively reviewed serologies of 76 infected total knee arthroplasty (TKA) patients who were treated with a two-stage exchange [5]. More recently, Bejon et al. came to a similar conclusion after analysing a dataset of 151 total joint arthroplasty patients (71 hip, 76 knee and four elbow revisions) who had undergone two-stage revision for PJI. They also analysed a dataset of 109 patients who had undergone DAIR (51 hip replacement, 50 knee replacements and eight other joints). They found that CRP had an AUC of 0.65 for predicting failure of DAIR at one year and concluded that CRP testing in this subgroup was of marginal usefulness [6].

Our aim was to examine the usefulness of CRP testing in determining whether a PJI has been treated successfully.

## Materials and methods

Three clinical datasets were retrospectively gathered from patients with PJI managed in a single tertiary referral centre specialising in treating PJI between April 2011 and March 2017. All cases treated with DAIR, single or two-stage revisions for PJI were included. Infection was diagnosed according to the Musculoskeletal Infection Society (MSIS) criteria [2]. All patients had been treated by the same surgical team. In all cases, patients had been treated with antibiotics for at least six weeks postoperatively according to organism sensitivity.

CRP results were collected preoperatively and at weeks one, three and six postoperatively. These results were collected from the electronic pathology reporting system. Clinical notes were reviewed for each patient to determine the organism responsible for PJI and to identify cases with persistent infection after treatment. The condition of persistent infection was defined based on the following criteria: (i) requiring further surgery to eradicate infection, (ii) presence of fever, rigors or purulent drainage postoperatively or (iii) chronic joint pain and swelling lasting 1-2 years postoperatively.

All data analysis was performed using Statistical Product and Service Solutions (SPSS) software version 25 (IBM, Armonk, NY) with significance set at a = 0.05. Mean values for CRP were calculated preoperatively and at weeks one, three and six postoperatively for DAIR, single-stage revision and two-stage revision respectively. Receiver operating characteristic (ROC) curves were calculated from mean CRP values. This allowed us to examine the area under the curve (AUC). The area under the curve determines how well a test separates the group being tested into those with and without the disease in question [7]. The traditional academic point system is a guide for determining the accuracy of a diagnostic test (Table 1).

**Table 1:** Traditional academic point system for AUC test values AUC: area under receiver operating characteristic curve

AUC value	Accuracy of diagnostic test
.90-1	Excellent
.80-.90	Good
.70-.80	Fair
.60-.70	Poor
.50-.60	Fail

There was no research-related contact with patients and all data was anonymised. Informed consent was waived for our study.

## Results

A total of 121 (hip 73, knee 48) cases were identified. Of these cases, 68 (hip 43, knee 25) had been treated with single-stage revision, 24 (hip 15, knee 9) with two-stage revision and 29 (hip 15, knee 14) with DAIR. The average age of the patient was 68 (range: 27-90) with 61 male patients and 60 female patients. PJI was found to be eradicated in 95 of the 121 patients (79%) in the cohort. There were 11 cases of persistent infection in 68 patients (16%) treated with single-stage revision. There were nine cases of persistent infection in 24 patients (38%) treated with two-stage revision. There were six cases of persistent infection in 29 patients (21%) treated with DAIR. 

There were 444 CRP results collected for 121 patients. There were 241 CRP results for 61 patients in the single-stage revision cohort (comprising 43 hip replacements and 25 knee replacements). There were 92 CRP results collected for 24 patients in the two-stage revision cohort (comprising 15 hip replacement and nine knee replacements). There were 111 CRP results collected for 29 patients in the DAIR cohort (comprising 15 hip replacements and 14 knee replacements).

Single-stage revision

**Figure 1:**
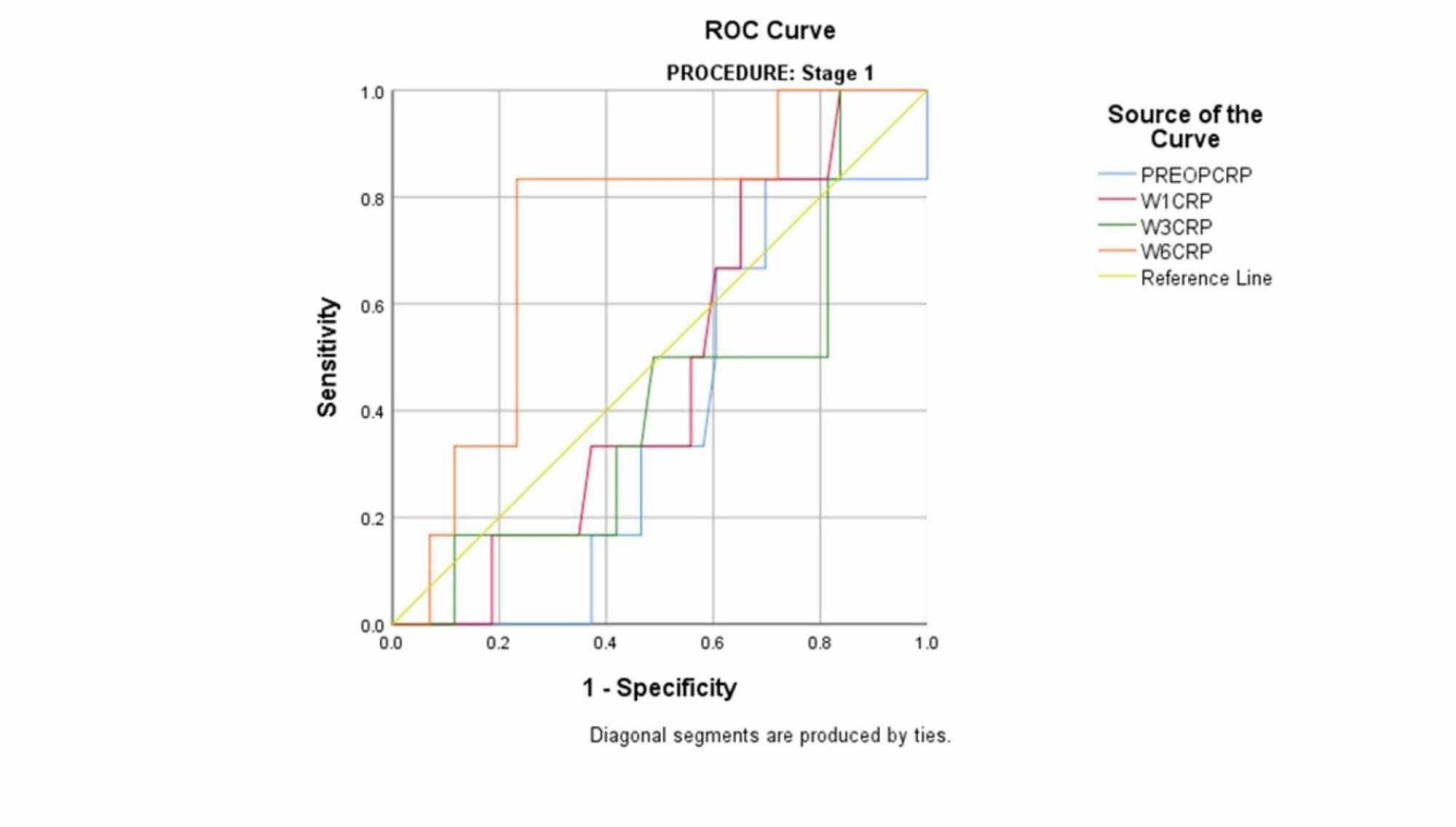
receiver operating characteristic curve for single-stage revision ROC: receiver operating characteristic PREOPCRP: preoperative C-reactive protein W1CRP: week-1 C-reactive protein W3CRP: week-3 C-reactive protein W6CRP: week-6 C-reactive protein

The mean preoperative CRP of the single-stage revision cohort was 62 [95% confidence interval (CI): 42-83]. One week postoperatively, the mean CRP of the cohort was 50 (95% CI: 40-59). At week three postoperatively, the mean was 43 (95% CI: 24-61). At week six postoperatively, the mean was 23.66 (95% CI: 15-33) (Table 2). There was no statistically significant difference between the mean CRP for patients who remained persistently infected and those who remained uninfected. ROC curves were produced using the mean CRP values of the cohort. The AUC values for weeks one and three were 0.471 and 0.421 respectively, indicating that testing at this time led to poor results in predicting reinfection (Figure 1) (Table 3). The AUC at week 6 was 0.733, indicating that this CRP testing was moderately useful; however, this test was not statistically significant (p: 0.67).

**Table 2:** Mean CRP values in single-stage revision cohort CRP: C-reactive protein

Inflammatory marker	Mean	95% confidence interval	Standard deviation
Preoperative CRP	62.26	41.95-82.56	65.99
Week-1 CRP	49.75	40.35-59.16	30.56
Week-3 CRP	42.79	24.42-61.16	59.70
Week-6 CRP	23.66	14.75-32.57	28.95

**Table 3:** Area under the curve values for single-stage revision CRP: C-reactive protein

Test result variable(s)^a^	Area	Std. error^b^	Asymptotic significance^c^	Asymptotic 95% confidence interval	
				Lower bound	Upper bound
Preoperative CRP	.378	.099	.337	.184	.572
Week-1 CRP	.471	.102	.819	.270	.671
Week-3 CRP	.421	.120	.532	.185	.657
Week-6 CRP	.733	.100	.067	.537	.928
a. Procedure = stage 1					
b. Under the nonparametric assumption					
c. Null hypothesis: true area = 0.5					

Two-stage revision

**Figure 2:**
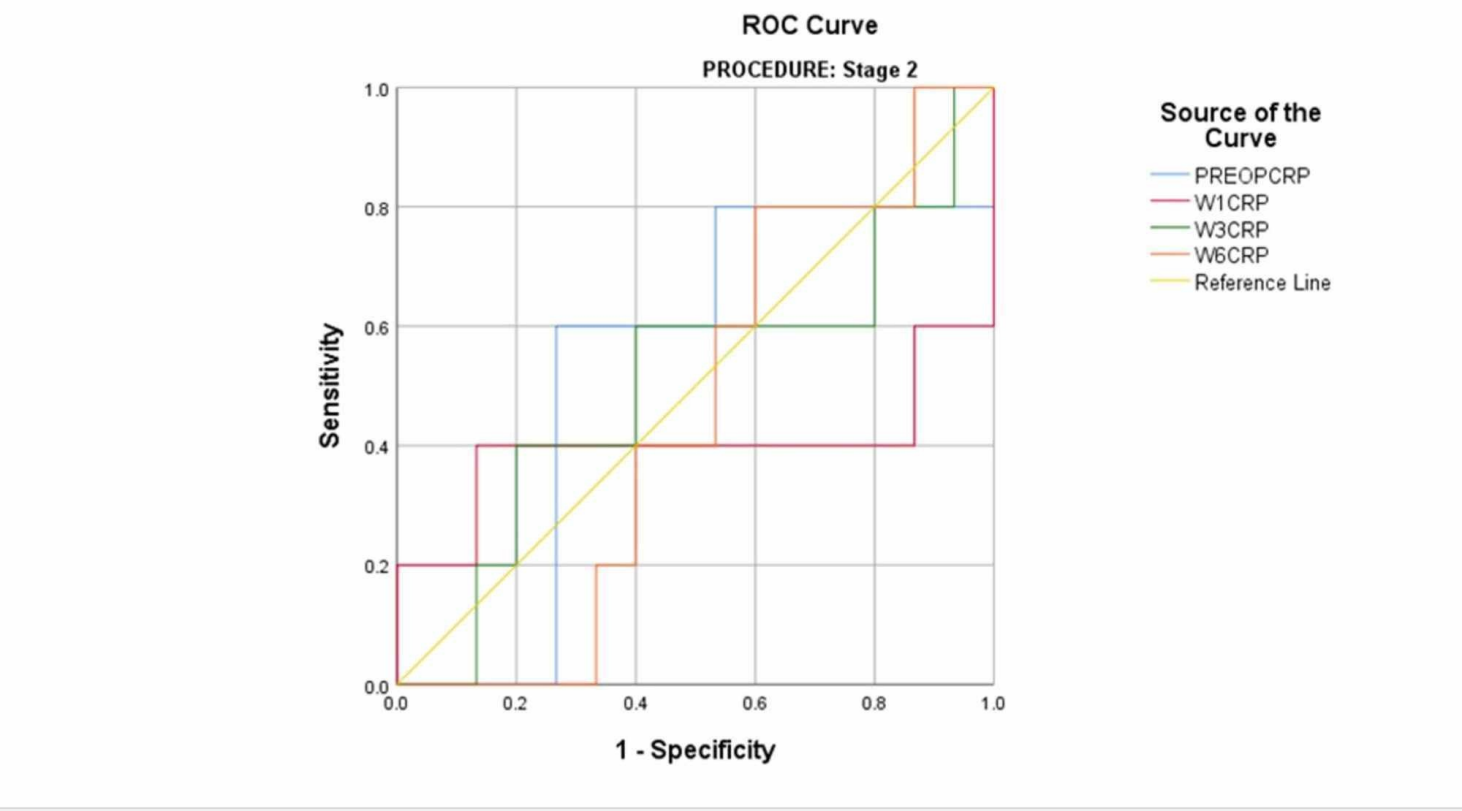
receiver operating characteristic curve for second-stage revision ROC: receiver operating characteristic PREOPCRP: preoperative C-reactive protein W1CRP: week-1 C-reactive protein W3CRP: week-3 C-reactive protein W6CRP: week-6 C-reactive protein

The mean preoperative CRP of the two-stage revision cohort was 63 (95% CI: 39-88). One week postoperatively, the mean CRP of the cohort was 60 (95% CI: 29-91). At week three postoperatively, the mean was 36 (95% CI: 18-53). At week six postoperatively, the mean was 23 (95% CI: 13-33) (Table 4). There was no statistically significant difference between the mean CRP for patients who remained persistently infected and those who remained uninfected. ROC curves were produced using the mean CRP values of the cohort. The AUC values for CRP testing in this cohort at all times were <0.6, indicating that the test was not useful (Figure 2) (Table 5).

**Table 4:** Mean CRP values in two-stage revision cohort CRP: C-reactive protein

Inflammatory marker	Mean	95% confidence interval	Standard deviation
Preoperative CRP	62.52	39.26-85.79	46.78
Week-1 CRP	59.90	28.79-91.01	62.56
Week-3 CRP	35.67	18.25-53.08	35.03
Week-6 CRP	22.98	12.60-33.35	20.86

**Table 5:** Area under the curve values for second-stage revision CRP: C-reactive protein

Test result variable(s)^a^	Area	Std. error^b^	Asymptotic significance^c^	Asymptotic 95% confidence interval	
				Lower bound	Upper bound
Preoperative CRP	.533	.156	.827	.227	.840
Week-1 CRP	.400	.200	.513	.008	.792
Week-3 CRP	.507	.162	.965	.188	.825
Week-6 CRP	.453	.132	.760	.194	.713
a. Procedure = stage 2					
b. Under the nonparametric assumption					
c. Null hypothesis: true area = 0.5					

Debridement, antibiotics and implant retention

**Figure 3:**
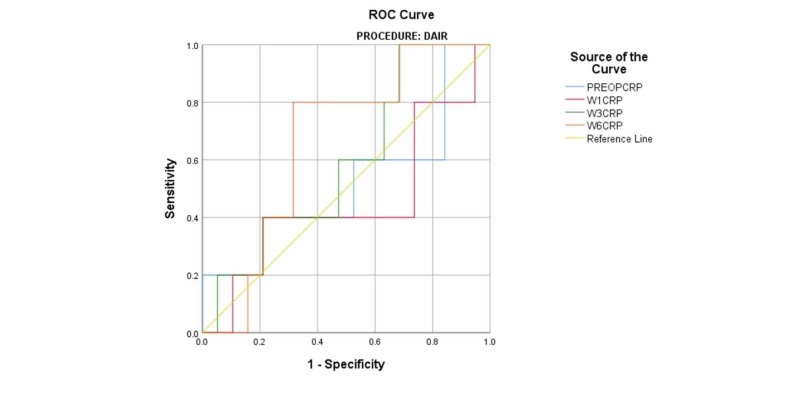
Receiver operator curve for DAIR ROC: receiver operating characteristic DAIR: debridement, antibiotics and implant retention PREOPCRP: preoperative C-reactive protein W1CRP: week-1 C-reactive protein W3CRP: week-3 C-reactive protein W6CRP: week-6 C-reactive protein

The mean pre-operative CRP of the DAIR cohort was 131 (95% CI: 86-177). One week postoperatively, the mean CRP of the cohort was 72 (95% CI: 51-94). At week three postoperatively, the mean was 59 (95% CI: 31-88). At week six post-operatively, the mean was 56 (95% CI: 21-91) (Table 6). There was no statistically significant difference between the mean CRP for patients who remained persistently infected and those who remained uninfected. ROC curves were produced using the mean CRP values of the cohort. The AUC values for CRP testing in this cohort at all times were <0.6, indicating that the test was not useful (Figure 3) (Table 7).

**Table 6:** Mean CRP values in DAIR cohort DAIR: debridement, antibiotics and implant retention CRP: C-reactive protein

Inflammatory marker	Mean	95% confidence interval	Standard deviation
Preoperative CRP	131.63	86.11-177.15	107.80
Week-1 CRP	72.16	50.54-93.79	51.21
Week-3 CRP	59.49	30.64-88.33	68.31
Week-6 CRP	56.36	22.13-90.60	81.10

**Table 7:** Area under the curve values for DAIR DAIR: debridement, antibiotics and implant retention CRP: C-reactive protein

Test result variable(s)^a^	Area	Std. error^b^	Asymptotic significance^c^	Asymptotic 95% confidence interval	
				Lower bound	Upper bound
Preoperative CRP	.516	.164	.915	.195	.837
Week-1 CRP	.453	.162	.749	.136	.769
Week-3 CRP	.589	.135	.546	.325	.854
Week-6 CRP	.663	.120	.271	.429	.897
a. Procedure = DAIR					
b. Under the nonparametric assumption					
c. Null hypothesis: true area = 0.5					

## Discussion

Based on our results, we could not recommend CRP as a test for determining the eradication of PJI. Our analysis generated low AUC values for all treatment modalities, indicating poor sensitivity and specificity of the test. This could potentially reflect the limited power of the study. However, it is more likely that the wide scatter of readings could be contributing to this. Treatment failure after DAIR, single-stage and two-stage revision was not associated with a high CRP at any measured time point. There was no statistical difference in mean CRP between patients with treatment failure and those who had successful treatment with any treatment modality.

PJI is a significant problem and carries a high morbidity rate for the patients. it is also becoming increasingly expensive to manage with a projected spend of US$ 1.62 billion in the US alone [8]. Research from Europe shows that the presence of infection triples the cost of a primary joint arthroplasty [9]. There have been many advances in surgical technique and preoperative diagnosis; however, there remains less clarity and consensus on the optimal criteria to assess response to treatment [2]. We feel it is essential to have a prognostic algorithm that allows for the use of inexpensive and readily available tests. Our research supports the current consensus that CRP in isolation is not useful in determining the eradication of infection. Recent studies have shown that interleukin-6 (IL-6) has a higher specificity for detecting the presence of infection; however, this test remains relatively expensive and inaccessible to the average surgeon [10,11].

We feel that more work needs to be done in reaching a consensus on how to monitor PJI treatment. A potential topic of further study would be to examine the usefulness of CRP in conjunction with other inflammatory markers such as white cell count and erythrocyte sedimentation rate.

## Conclusions

Our study found that serial CRP testing was not a reliable test for determining the eradication of PJI in cases treated with single-stage revision, two-stage revision or DAIR. We feel that more work needs to be done to establish a widely accepted and reliable algorithm to determine the resolution of the infection.
